# Perceptions and practices of UK GPs towards youth vaping: a questionnaire-based study

**DOI:** 10.3399/BJGPO.2025.0080

**Published:** 2026-01-28

**Authors:** Elika Najafi, Devan Wasan, Yasmin Baker, Kristian Peters, Dhruv Vasooja, Maneth Warnapala, Mario Martínez-Jiménez

**Affiliations:** 1 Imperial Business School, Imperial College London, London, UK; 2 University of Manchester School of Medicine, University of Manchester, Manchester, UK; 3 Imperial College School of Medicine, Imperial College London, London, UK; 4 Barts and The London School of Medicine and Dentistry, Queen Mary University of London, London, UK; 5 Department of Health Policy, Stanford University, Stanford, California, United States; 6 Department of Economics and Public Policy, Imperial Business School, London, UK; 7 Centre for Health Economics and Policy Innovation, and Centre for Paediatrics and Child Health, Imperial College London, London, UK

**Keywords:** general practice, primary health care, vaping, adolescent, young adult

## Abstract

**Background:**

E-cigarette use among adolescents and young adults is a growing public health concern. GPs play a critical role in addressing health behaviours, yet there is limited research on their perceptions and practices towards vaping in young people.

**Aim:**

To explore current perceptions and practices among GPs concerning vaping in young people.

**Design & setting:**

A quantitative approach was employed through an online, self-administered questionnaire. Responders included both trainee and qualified GPs from across the UK.

**Method:**

The questionnaire consisted of Likert-scale and free-text questions, covering screening, counselling, and demographic information. Participants were recruited through newsletters and social media, with 284 responses collected from March 2024 to August 2024. Data were analysed using descriptive statistics. The Kruskal–Wallis test was used to assess for significant differences based on responders’ region of work or level of experience.

**Results:**

GPs rarely enquire about e-cigarette use (23.9%), despite 85.6% believing it is important to do so. Lack of time, relevance to the presenting complaint, and method for quantifying and documentation were cited as factors contributing to low screening rates. Only 27.5% of GPs provide advice to e-cigarette users, likely owing to low rates of confidence (12.3%). A lack of time, understanding of health effects, training, and availability of referral services were cited as factors contributing to low counselling rates. There were no significant variations in responses based on responders' location or level of experience.

**Conclusion:**

GPs recognise the importance of youth vaping but face barriers to screening and counselling, indicating the need for change in guidelines and policy.

## How this fits in

The rise of youth vaping has not been adequately explored within primary care, particularly in the UK, where existing literature primarily focuses on non-primary care interventions. Current discussions tend to view vaping merely as a smoking cessation tool, neglecting its distinct presence among youth populations, which exacerbates the lack of evidence on how GPs can address this behaviour. Our study contributes valuable insights by gathering primary data on GPs' perspectives and practices, identifying both barriers and opportunities for intervention. These findings provide a foundation for developing evidence-based strategies to improve screening and counselling efforts in primary care settings.

## Introduction

The prevalence of e-cigarette use, or 'vaping', among young individuals has significantly increased, from 3.8% in 2013 to 20% in 2023.^1^ In 2024, approximately 40% of children aged 11–17 years who experimented with vaping had never previously smoked conventional cigarettes.^1^ This phenomenon is far less pronounced in adults, with only 8% of e-cigarette users reporting having never smoked traditional cigarettes.^2^ This study aims to explore GPs' perceptions and practices concerning e-cigarette use among youth populations, aiming to better understand their role in addressing this increasingly troubling issue.

Vaping had initially been hailed as ‘*95% safer than smoking*’,^3^ but new research has raised concerns about its biological, psychological, and social health implications. The contents of e-cigarettes have the potential for harm and lung injury, with ingredients including *‘cancer-causing chemicals’*, heavy metals, and chemical flavourings that have been linked to serious lung diseases such as popcorn lung.^4^ Use has also been linked to exacerbation of pre-existing respiratory conditions such as asthma and chronic obstructive pulmonary disease (COPD).^4,5^

While evidence relating to the health impacts of vaping is limited, stronger evidence exists regarding the psychological impacts, primarily owing to the high volumes of nicotine within these products, with the highest concentrations often found in devices used by young adults.^6^ E-cigarette use often begins as a way to cope with psychological distress, low mood, and social isolation. While it provides immediate stress relief, it can lead to nicotine dependence, worsening the cycle of psychological stress.^7^ Other research supports these findings, indicating that nicotine is associated with poorer self-rated health, higher stress levels, and potentially negative effects on academic performance.^8,9^

Moreover, there are concerns regarding the potential ‘gateway effect’ linked to vaping among young people. Research indicates that vaping may be linked to the initiation of conventional smoking, marijuana smoking, and alcohol abuse, raising significant healthcare concerns within this demographic.^10,11^ Additionally, a US study found that four in 10 young people who vape alter their e-cigarettes in ways not intended by the manufacturers. These modifications frequently involve adding unregulated refillable pods and incorporating illicit substances such as marijuana.^12^ Clearly, reducing usage is important for young people who do not smoke tobacco, especially where minimising harm from traditional cigarette smoking is not a priority.

GPs play a pivotal role in the healthcare system, with the Department of Health and Social Care stating that ‘*more than 90% of a patient’s direct experience of the NHS is through primary care*’.^13^ GPs are regularly updated with important information, enabling them to address new health challenges. Schemes such as Very Brief Interventions (VBI) and Making Every Contact Count (MECC), which aim to tackle unhealthy behaviours, can be significantly enhanced by the expertise and rapport of the GP.^14^ Thus, GPs are well-suited to tackle the rising concern of youth vaping. Consequently, this article examines the research question: what are GPs' views and practices regarding e-cigarette usage among young people (≤24 years old) in the UK?

## Method

### Study design

The study was a cross-sectional survey of GPs in the UK conducted from March 2024 to August 2024. The survey was developed and piloted with three GPs. Feedback from the pilot phase led to revisions of the questionnaire, with participating GPs assessing the relevance of each question and the likelihood of any question miscomprehension.

The questionnaire was divided into the following three sections: screening and recording practices; counselling and cessation practices; and demographic information for stratifying responders by region and experience. The full list of survey questions and response options can be found in Appendix A.

### Population and setting

The target population comprised all primary care doctors in the UK, including GP trainees. Participants were recruited through self-selection. The available population for sampling was estimated to be approximately 56 079.^15–18^ The questionnaire was disseminated through university primary care departments’ newsletters and professional social media platforms such as LinkedIn.^19^

### Data analysis

The online platform Qualtrics^20^ was used for questionnaire distribution and data collection. Responses were analysed using R Studio.^21^ The software summarised quantitative data using descriptive statistics, while free-text responses were categorised into thematic groups.

Variation in responses was examined by region and experience using the Kruskal–Wallis test to determine statistical significance. For this analysis, ‘experience’ was grouped into GP specialty trainees (GPSTs), 0–5 years, 6–10 years, 11–15 years, 16–20 years, 21–25 years, 26–30 years, and >31 years.

### Outcomes

The primary outcomes of this study were to examine the screening and counselling practices of GPs regarding e-cigarette use among young people. This includes examining how often GPs ask about e-cigarette use and the extent of their advice on health risks. Secondary outcomes focused on identifying barriers to effective screening and counselling, as well as exploring any variation in practices based on GPs' years of experience and regional location.

## Results

**Figure 1.**
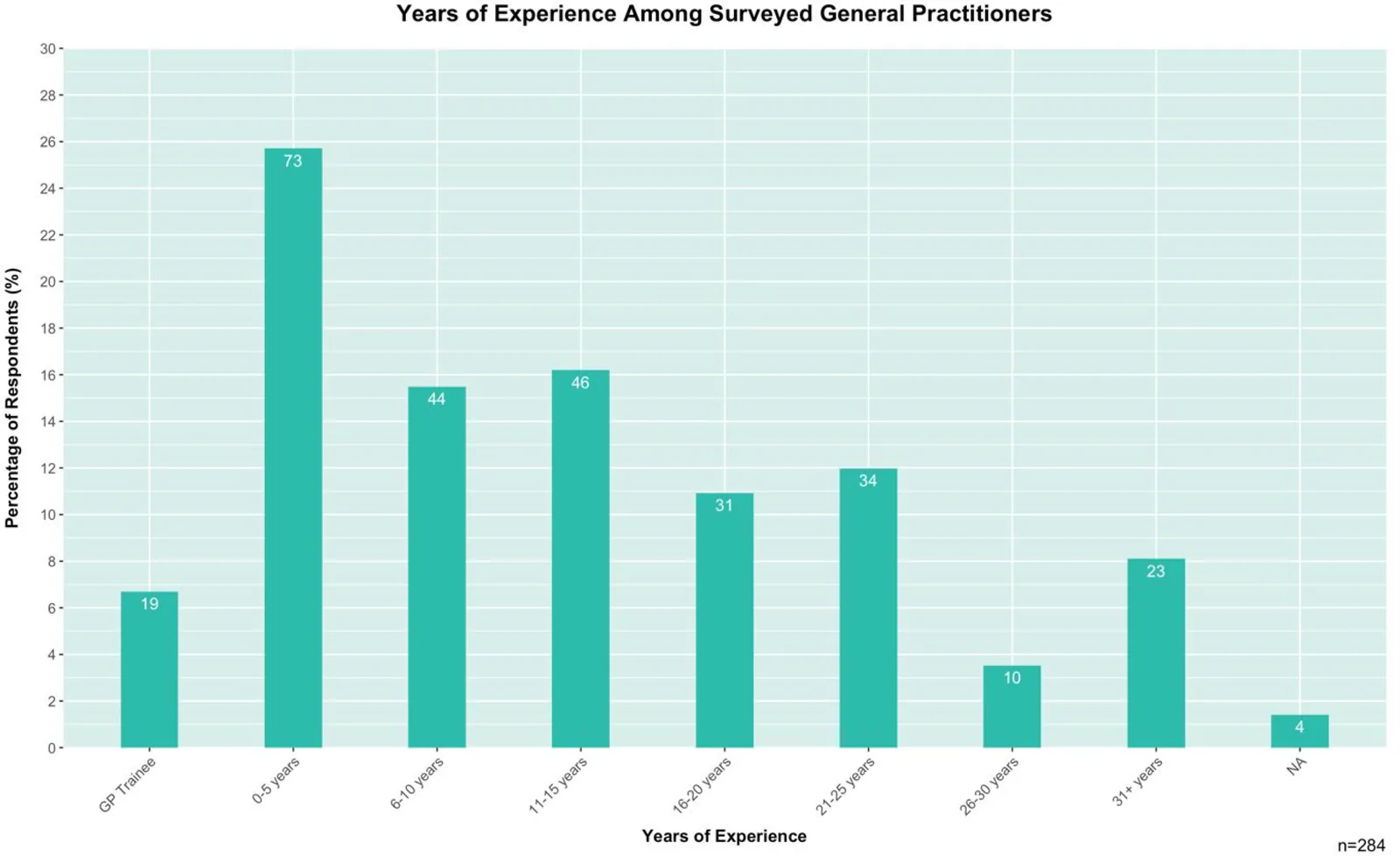
Surveyed GPs' years of experience

Over 6 months, from March 2024 to August 2024, 284 completed questionnaires were collected. With a 95% confidence level, this sample size provides a margin of error of 6% (based on a population size of 56 079 GPs).^15–18^ Figure 1 illustrates the distribution of GPs based on their years of experience.

**Figure 2.**
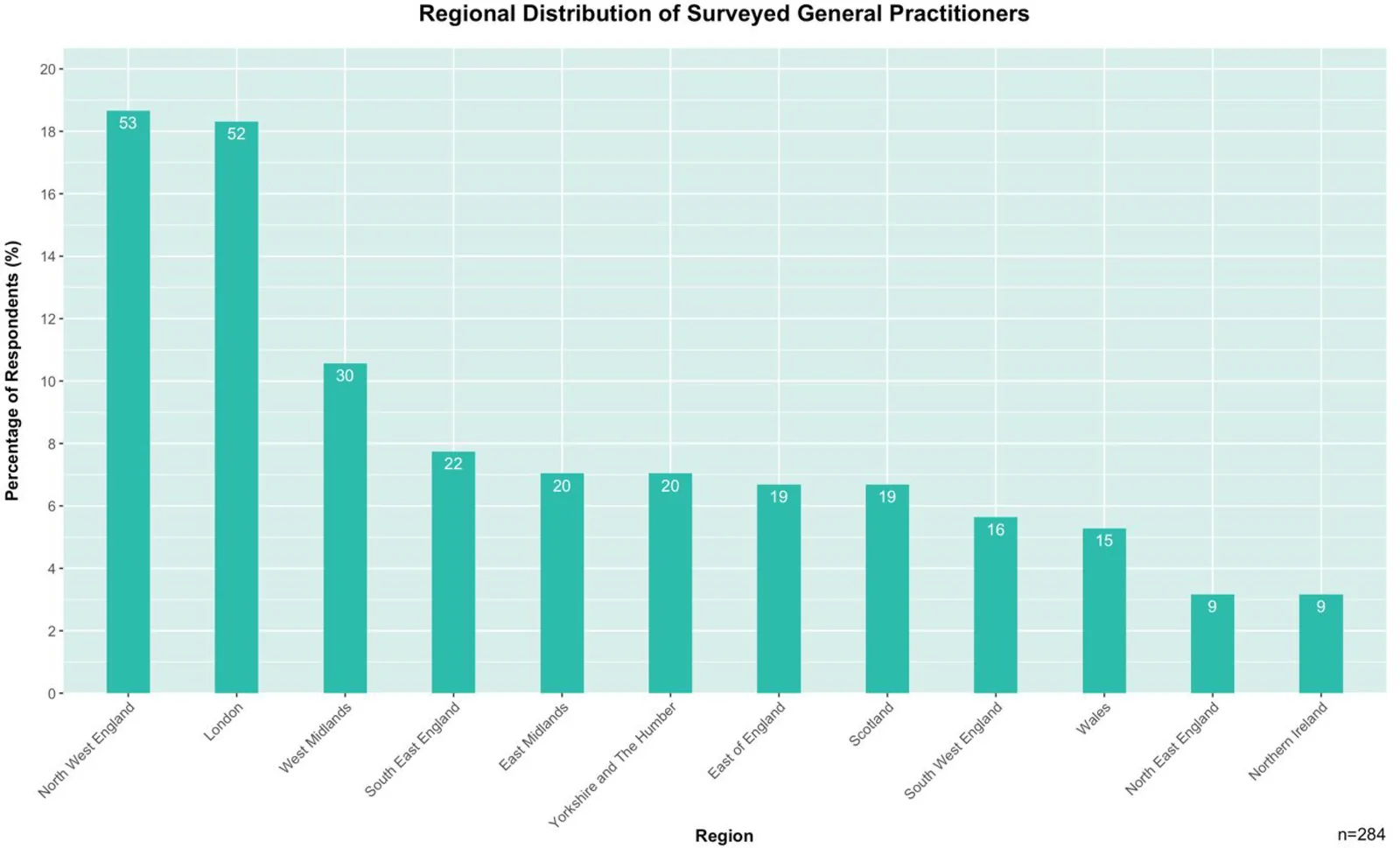
Surveyed GPs' region of work

Most responders worked in densely populated regions of the UK (London and the North-West of England). Figure 2 illustrates the total number of responders from each region of the UK. A comparison of the sampled population demographics with UK data is available in Appendix B.

### Screening and recording

GPs infrequently enquire about the e-cigarette use of their adolescent patients during consultations, with only 23.9% indicating that they do so consistently or frequently. This is noteworthy given that a substantial 85.6% of responders consider it important to investigate e-cigarette usage, independent of cigarette smoking status (Table 1) .

**Table 1.** Responses to questions relating to screening practices

Question	Response	Percentage	Frequency
How frequently do you ask adolescent and young adult patients about their e-cigarette status during consultations?	All or most of the time	23.9%	68
	Sometimes	45.4%	129
	Rarely or never	30.6%	87
How important do you think it is to enquire about adolescents, and young adults‚ e-cigarette status, separate from smoking status?	Very important	50.7%	144
	Slightly important	34.9%	99
	Neutral	10.9%	31
	Slightly not important	2.5%	7
	Not important	1.1%	3

**Figure 3.**
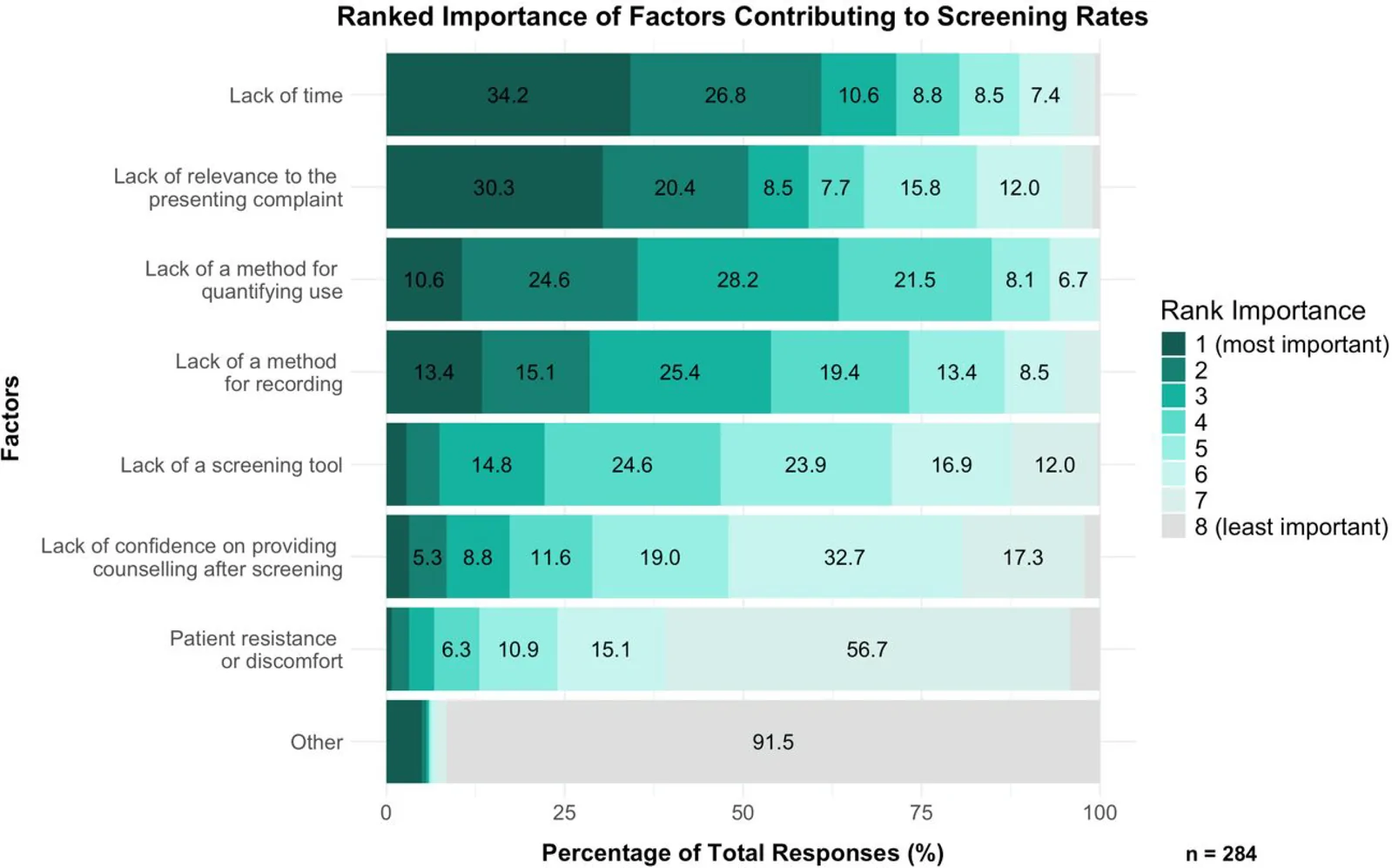
Implicated factors pertaining to low screening rates

When participants were prompted to rank various factors contributing to low screening rates according to their significance, the four most critical factors identified were, in descending order of importance: lack of time, lack of relevance to the presenting complaint, absence of a method for quantifying utilisation, and the lack of a systematic approach for documentation (Figure 3).

These findings highlight the potential challenges within primary care that must be considered when developing and implementing recommendations.

In the ‘other’ section, participants provided free-text responses regarding factors they believe hinder screening for e-cigarette use within this population (*n* = 36). The predominant theme identified was insufficient engagement with adolescents. Responders reported that adolescents attend infrequently, and when they do so, they are often accompanied by parents, complicating the initiation of discussions around sensitive topics such as e-cigarette use. One GP noted, *‘Teenagers rarely go to the GP*,’ while another remarked, ‘*They tend to attend with parents, making it challenging to discuss personal issues like smoking*.’

The second most prominent theme was the uncertainty surrounding the potential health consequences of vaping. Some GPs conveyed that e-cigarettes are often perceived as safer alternatives to traditional cigarettes, which complicates discussions about their usage among younger populations. Practitioners also pointed out the challenges in addressing this topic owing to the absence of definitive evidence. One GP remarked, *‘Teenagers perceive e-cigarettes as less harmful than smoking, thereby creating a barrier to productive discussions*.’ Another GP commented, ‘*There is a lack of rigorous data regarding the health effects of e-cigarettes, which hampers our ability to provide informed guidance.*’

### Counselling and cessation

GPs infrequently provide guidance to young e-cigarette users regarding the health risks associated with vaping. Only 27.5% of surveyed practitioners reported advising their patients on this topic during consultations (Table 2) . This is important, as practitioners are the most likely to initiate discussions on this topic during consultations, given that a reported 90.5% of young e-cigarette users rarely or never seek advice. Responders indicated a lack of confidence in their understanding of e-cigarettes as a reason for this, particularly concerning their potential health impacts. Only 12.3% of participants expressed high levels of confidence in their knowledge of this issue; 46.8% were somewhat confident, while 40.8% reported no confidence at all (Table 2).

**Table 2.** Responses to questions relating to counselling practices

Question	Response	Percentage	Frequency
How frequently do adolescent and young adult patients ask for advice regarding e-cigarette use?	All or most of the time	1.8%	5
	Sometimes	7.7%	22
	Rarely or never	90.5%	257
If a patient is an e-cigarette user, how frequently do you actively advise or inform them about the health impacts of using e-cigarettes during consultations?	All or most of the time	27.5%	78
	Sometimes	43.0%	122
	Rarely or never	30.0%	84
How confident do you feel in your knowledge about the health effects of e-cigarette use?	Very confident	12.3%	35
	Somewhat confident	46.8%	133
	Not confident at all	40.8%	116

**Figure 4.**
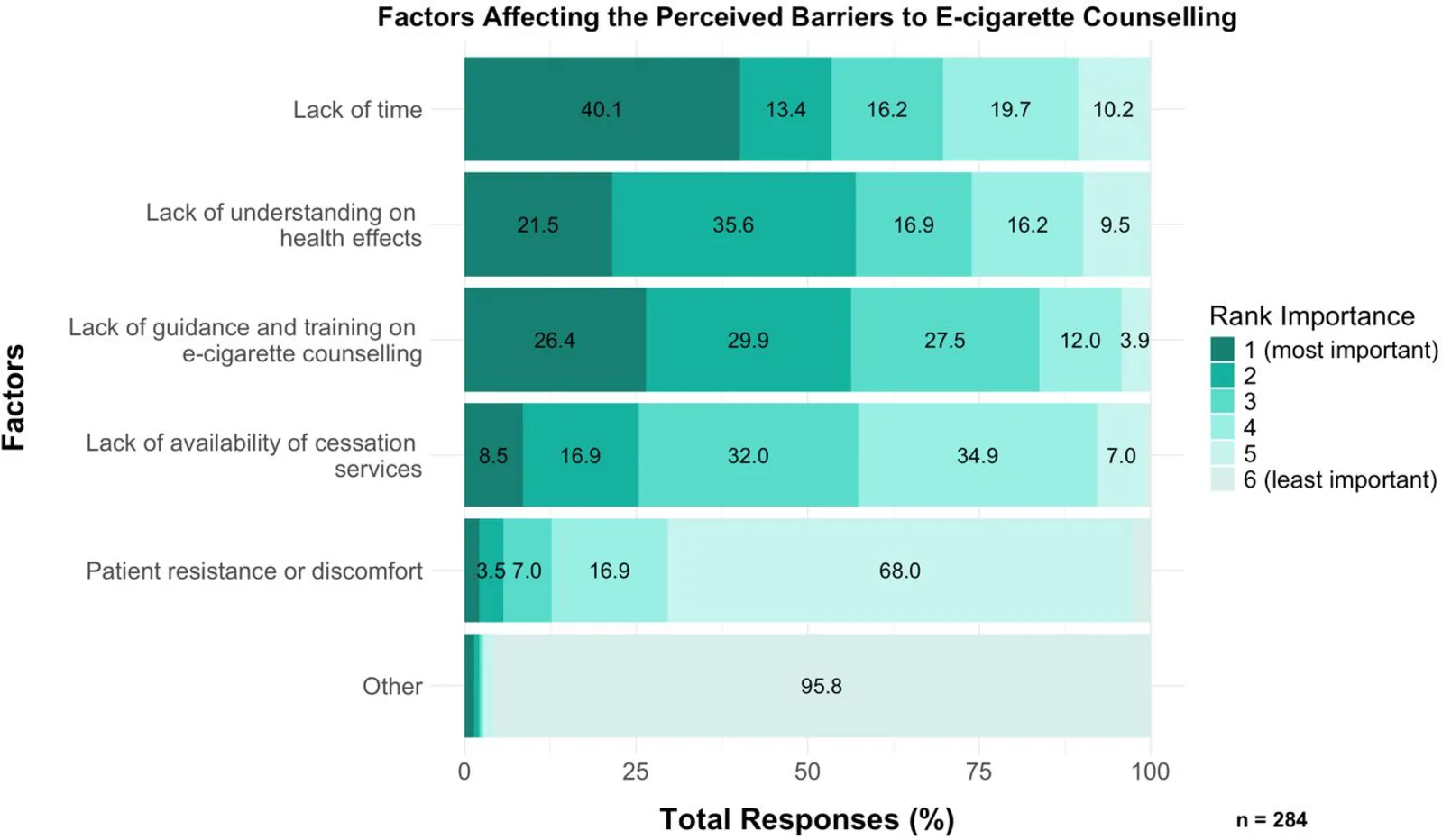
Implicated factors pertaining to low counselling rates

When asked about possible factors contributing to such low counselling rates, once again, a lack of time was cited as the most important factor, followed by a lack of understanding of health effects, a lack of guidance and training, and a lack of availability of referral services (Figure 4).

In the free-text responses (*n* = 10) categorised under ‘other,’ the predominant theme identified was insufficient knowledge or confidence among GPs. Responders reported unfamiliarity with e-cigarette products, including brands, strengths, and health effects. Furthermore, the comparison between e-cigarettes and conventional cigarettes emerged as a significant concern. Notably, it was mentioned that *‘balancing the effects of e-cigarettes versus cigarettes is challenging*’. Additionally, the scarcity of rigorous data presented an impediment to a comprehensive understanding of the subject.

No significant associations were found between the region where responders worked or their years of experience and their responses (Appendix C, Table C1). This suggests that the findings are not specific to particular regions or experience levels, suggesting that the issues of low screening and counselling may be widespread.

## Discussion

### Summary

GPs showed a noteworthy intention–behaviour gap regarding youth vaping, with 85.6% believing screening is important, but only 23.9% actually doing it. Barriers to screening include a lack of time, absence of systematic documentation methods, and limited engagement with adolescent patients, who often attend with parents. Additionally, GPs expressed uncertainty regarding the health effects of vaping, citing insufficient data and perceived safety compared with traditional smoking as complicating factors. A similar gap in belief and behaviour was displayed with counselling behaviours, with only 27.5% of GPs advising young patients on its health risks. Confidence in understanding the health impacts of e-cigarettes was low (12.3%), and barriers again included lack of time, understanding of health effects and guidance, and referral services. These challenges appeared consistent across regions and experience levels, suggesting systemic issues.

### Strengths and limitations

This is the first cross-sectional study from the UK aimed at understanding the perceptions and practices of GPs regarding vaping in the young non-smoking population. Conducting a quantitative review with a substantial sample size on this topic, utilising data derived from demographically diverse populations across the UK, significantly enhances our understanding of these perceptions and practices. However, the collected demographic data (years of experience and location) was not statistically representative of the UK population, as shown in Appendix B. The absence of further demographic comparisons further limits the generalisability of our results.

Initially, we preferred to use years of experience as a demographic metric rather than age, recognising that some doctors may graduate later or take breaks during their careers. However, national statistics are only reported by age; thus, we used years of experience as a proxy for age by adding 26 years, which is the minimum age at which a doctor can start GP training. This conversion method may lead to a systematic underestimation of age. Moreover, the sampling methodology, particularly through primary care newsletters, may introduce a degree of self-selection bias; it is plausible that GPs who are particularly committed to this topic or population have disproportionately chosen to participate in the study.

### Comparison with existing literature

This study builds on the findings of Singh *et al* (2024),^22^ which found that 55% of GPs lacked a structured approach to screening and counselling young patients, and 45% found these conversations to be ‘*difficult*’. The study identified a lack of knowledge, confidence, and structured discussion methods as key contributors to the issue, consistent with our findings. Singh *et al* also highlight challenges with access to younger patient populations, awareness of patients using e-cigarettes who were otherwise non-smoking, and the specific time constraints present in the NHS. This insight enables clinicians and policymakers in the UK to formulate practical recommendations aimed at enhancing practices.

Further research in America by Becker and Rice (2022)^23^ highlights a lack of screening tools and uncertainty around long-term health impacts as two primary causes of low screening and counselling rates, respectively. While it is important to consider the differences between the American and British healthcare systems, this article highlights discussion points for counselling young patients, for example, by highlighting the adverse health effects that may be associated with e-cigarette use.

Parallels can be drawn from research conducted by Selamoglu *et al* (2022),^24^ which examined the knowledge, perceptions, and attitudes of GPs towards e-cigarettes in the context of smoking cessation. This research further suggests that GPs feel ill-equipped to have discussions about e-cigarettes with patients. Notably, both our study and this systematic review highlight a lack of training, inconsistent knowledge, and uncertainty regarding safety as barriers to effective patient counselling. Interestingly, GPs in this systematic review express concern over the rising popularity of e-cigarettes among our demographic, fearing that they may serve as a gateway to smoking.^24^

While training and evidence-based recommendations may take time to emerge, useful research has been published by White *et al* (2023),^25^ which offers advice for GPs to consider when discussing e-cigarette use with young patients. For example, they highlight the importance of routinely asking and recording e-cigarette use as part of the Home, Education/employment, Eating/exercise, Activities, Drugs, Sexuality, Suicide/depression, and Safety (HEADSS) assessment. A comprehensive history should follow this if e-cigarette use is identified. The authors also suggest that GPs should explore patients' understanding of the health impacts and stress the environmental impacts of vaping, which can serve as a motivational tool.^25^ While such suggestions provide excellent starting points, this study emphasises the importance of considering time pressures when designing further interventions.

Lessons can be learnt from the smoking cessation drive of the 2000s when developing vaping cessation strategies, particularly considering the success of interventions in lowering smoking rates in the UK.^26^ A multi-country study by Stead *et al* (2009)^27^ suggests that postgraduate training and financial incentives are crucial for increasing GP involvement in smoking cessation efforts. The study also suggests that familiarity with local smoking cessation services boosts GPs' confidence in discussing smoking cessation.^27^ These findings are important for creating strategies to improve e-cigarette practices; however, the authors note that smoking interventions are more effective when smoking is linked to the presenting complaint. This may not apply to e-cigarette users, especially considering their younger demographics, infrequent visits to GPs, and a lack of conclusive evidence on e-cigarette health effects.

This study aligns with existing literature, highlighting GPs’ concerns surrounding youth vaping, with many feeling ill-equipped to address it. While lessons can be drawn from the smoking epidemic, they should be contextualised to the unique characteristics of today’s youth and the ambiguity of evidence surrounding the health impacts of vaping. Moreover, this study validates similar findings from the UK and underscores the need for strategies that consider the pressures currently faced by GPs.

### Implications for practice

This research highlights that GPs recognise youth vaping as an issue requiring their attention. Yet, they lack the confidence to act, leading to a clear need for improved screening and counselling practices regarding youth vaping. Our analysis concurrently identifies considerable barriers to and opportunities for the enhancement of these practices. The success of such interventions will depend on the implementation of evidence-based guidelines and educational resources for GPs. Additionally, policymakers may find it encouraging that GPs express a desire for these resources and are likely to welcome their development. The aim of these materials should be to equip GPs with effective tools for discussing e-cigarette use, particularly in consultations with young patients.

## Data

The dataset relied on in this article is available from the corresponding author on reasonable request.
